# Clearing the Air on Pollutant Disruptions of the Gut–Brain Axis: Developmental Exposure to Benzo[a]pyrene Disturbs Zebrafish Behavior and the Gut Microbiome in Adults and Subsequent Generations

**DOI:** 10.3390/toxics13010010

**Published:** 2024-12-25

**Authors:** Alexandra Alexiev, Ebony Stretch, Kristin D. Kasschau, Lindsay B. Wilson, Lisa Truong, Robyn L. Tanguay, Thomas J. Sharpton

**Affiliations:** 1 Department of Microbiology, Oregon State University, Corvallis, OR 97333, USA; stretche@oregonstate.edu (E.S.); kristin.kasschau@oregonstate.edu (K.D.K.); thomas.sharpton@oregonstate.edu (T.J.S.); 2 Department of Environmental and Molecular Toxicology, Oregon State University, Corvallis, OR 97333, USA; lindsay.wilson@doane.edu (L.B.W.); lisa.truong@oregonstate.edu (L.T.); robyn.tanguay@oregonstate.edu (R.L.T.); 3 Sinnhuber Aquatic Research Laboratory (SARL), Oregon State University, Corvallis, OR 97333, USA; 4 Department of Statistics, Oregon State University, Corvallis, OR 97333, USA

**Keywords:** zebrafish, development, neurotoxicity, benzo[a]pyrene, gut microbiome, behavior

## Abstract

Developmental exposure to benzo[a]pyrene (BaP), a ubiquitous environmental pollutant, has been linked to various toxic effects, including multigenerational behavioral impairment. While the specific mechanisms driving BaP neurotoxicity are not fully understood, recent work highlights two important determinants of developmental BaP neurotoxicity: (1) the aryl hydrocarbon receptor (AHR), which induces host metabolism of BaP, and (2) the gut microbiome, which may interact with BaP to affect its metabolism, or be perturbed by BaP to disrupt the gut–brain axis. We utilized the zebrafish model to explore the role of AHR, the gut microbiome, and their interaction, on BaP-induced neurotoxicity. We tested (1) how developmental BaP exposure and AHR2 perturbation in zebrafish link to adult behavior, (2) how these variables associate with the structure and function of the adult zebrafish gut metagenome, and (3) whether these associations are multigenerational. Our findings reveal a reticulated axis of association between BaP exposure, developmental AHR2 expression, the zebrafish gut metagenome, and behavior. Results indicate that AHR2 is a key modulator of how BaP elicits neurotoxicity and microbiome dysbiosis. Additionally, this axis of association manifests generationally. These findings demonstrate the importance of studying pollutant–microbiome interactions and elucidate the role of specific host genes in neurotoxicity and dysbiosis.

## 1. Introduction

Benzo[a]pyrene (BaP) is a model environmental chemical toxicant that is often used to study the effect of chemical exposure on organismal health. This is due to its ubiquity in the environment, impact on the early-life development of vertebrates, and ability to elicit such effects across multiple generations [1,2,3,4]. Well-known as a pro-carcinogen, studies have linked BaP to neurodevelopmental and neurodegenerative disorders [5,6,7,8,9,10,11]. Experiments in vertebrate model systems, notably zebrafish, support these observations, finding that developmental BaP exposure drives neurodevelopmental and neurodegenerative phenotypes [3,4,6,12]. These neurotoxic effects also appear to manifest in subsequent, unexposed generations [4,13], indicating that behavioral impairments caused by developmental BaP exposure elicit multigenerational effects. These observations fuel the effort to resolve the mechanisms that underlie BaP neurotoxicity, which currently remain poorly understood. Recent work points to two possible variables that may play a role in defining how developmental BaP exposure ultimately gives rise to behavioral impairments later in life: the gut microbiome and the aryl hydrocarbon receptor (AHR).

The gut microbiome may be an important component of its host’s biology that interacts with various chemical exposures, and researchers are just beginning to consider this component as part of the host response to toxicants [14,15,16,17,18,19,20,21,22,23,24]. The gut microbiome coordinates with the central nervous system through a reticulated axis of communication that is collectively referred to as the gut–brain axis [14,25,26,27,28,29]. The establishment of dysbiotic gut microbial communities has been shown to drive neurodegenerative phenotypes in mice and humans [30,31,32] due to perturbations to the homeostatic contribution of the gut microbiome to neurophysiology. Early-life exposures to environmental toxicants are theorized to perturb gut microbiome assembly and drive the successional development of dysbiotic communities. Indeed, our prior work found that early-life exposure to BaP perturbed gut microbiome assembly in zebrafish larvae [33]. However, it remains unknown if these effects ultimately manifest as differential microbial communities among developed (adult) fish, and if such effects persist in subsequent generations.

While the AHR pathway is a known mechanism underlying BaP genotoxicity, its role in BaP neurotoxicity remains poorly defined and of interest [7]. Recent research using AHR mutant zebrafish suggests that the receptor plays a role in larval hyperactivity resulting from early-life BaP exposure [3]. The AHR binds BaP directly, which downregulates AHR activity, as part of the regular host–toxicant response, and in doing so, initiates host metabolic pathways that biotransform BaP into catabolites that are themselves neurotoxic [2,7,34,35,36,37,38]. Moreover, the AHR is a mechanism through which hosts and their gut microbiota interact: the AHR binds gut microbial metabolites like tryptophan, inducing subsequent shifts in gut microbiome composition [19,36,39,40,41,42,43]. These observations raise the possibility that AHR may be a critical determinant of how BaP elicits neurotoxicity and, further, may interact with the gut microbiome in ways that affect this neurotoxic response. To our knowledge, no work to date has considered the effect of the AHR on the gut microbiome and neurotoxicity in the context of developmental BaP exposure.

**Figure 1:**
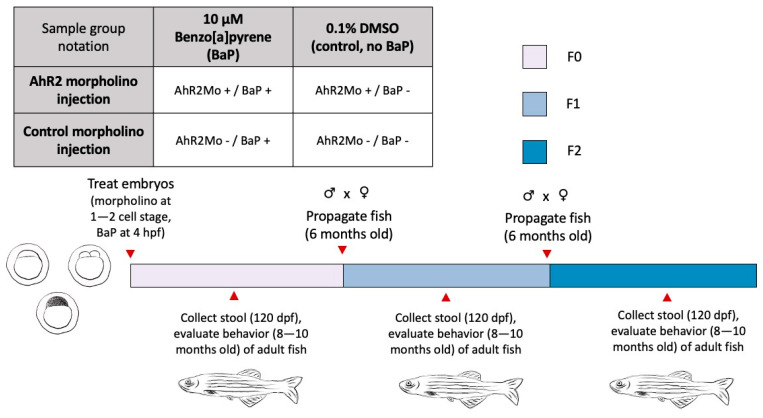
The factorial experimental design and workflow used in this study. The table at the top left shows the sample groups representing the treatments we applied to zebrafish embryos in the F0 generation. The notation in the table is what is used in the graphs and in the text throughout the paper. The bar represents the workflow chronology, with colors showing each generation and red arrows with text for relevant procedures and collections, and ages of fish throughout.

In this study, we sought to evaluate whether the AHR pathway and gut microbiome are linked to BaP neurotoxicity and its generational effects. To do so, we leveraged the zebrafish model, which affords a tractable and scalable means of controlling embryonic exposure, and specifically evaluated the gut microbiome and behavior of fish (F0) developmentally exposed to BaP, as well as their offspring (F1) and one subsequent generation (F2). Because zebrafish grow rapidly and have optically clear embryos, they are amenable to studies that investigate the developmental and generational consequences of embryonic exposure. The ability to rear large sample sizes is also of benefit, especially for microbiome work [24]. Although human behavior is far more complex than that of zebrafish, they are an adequate animal model for anxiety and social behaviors [3,4,44]. Additionally, morpholino technologies have been developed that enable the manipulation of specific zebrafish genes precisely only during the exposure period [45]. Here, we utilized a morpholino of the AHR2 gene to transiently knock down embryonic expression during development in F0 generation fish, using a dosage and protocol previously defined. This manipulation was concurrent with exposure to 10 µM of BaP, which our prior work found was sufficient to elicit neurodevelopmental and generational effects in zebrafish [4]. Thus, AHR2 function was only perturbed during exposure, and not later in the F0 generation’s life cycle or in subsequent generations. Specifically, we exposed developing zebrafish embryos to either 10 µM BaP (AHR2Mo-/BaP+), a morpholino that transiently blocks developmental AHR2 expression (AHR2Mo+/BaP-), both the toxicant and morpholino (AHR2Mo+/BaP+), or the relative controls (AHR2Mo-/BaP-) (Figure 1). Fish from all four treatments were then raised in chemical-free water to assess potential developmental effects (in adult F0 fish). We also evaluated effects in adult F1 and F2 fish, where the F1 generation represents an intergenerational mechanism and F2 represents a transgenerational mechanism in zebrafish. The analysis uncovered a link between BaP exposure, microbiome composition and functional capacity, and zebrafish behavior, and found that AHR2 was a key mediator of these associations. The analysis further indicates that the effect of developmental BaP or AHR2 morpholino exposure affects the microbiome and behavior of subsequent generations. Ultimately, this study supports the hypothesis that the gut microbiome and AHR pathways are components underlying BaP’s mechanisms of developmental and generational neurotoxicity.

## 2. Materials and Methods

### 2.1. Overview

To investigate the role AHR and the gut microbiome play in BaP neurotoxicity, embryonic zebrafish had their AHR2 gene transiently knocked down using a morpholino injection (AHR2Mo+) or were alternatively injected with a control morpholino (AHR2Mo-) (Figure 1). At 4 hpf, fish from these populations were either exposed to 0 µM BaP (AHR2Mo+/BaP- and AHR2Mo-/BaP-) or 10 µM of BaP (AHR2Mo+/BaP+ and AHR2Mo-/BaP+). Our previous study on the effect of various doses of BaP on zebrafish gut microbiota and behavior found that 10 µM was sufficient to elicit neurodevelopmental and generational effects in zebrafish [4]. Embryos were statically exposed to BaP in individual wells of a 96-well plate until 120 hpf, at which point the larvae were transferred to chemical-free water and raised until adulthood (F0) before undergoing adult behavioral assessments and gut microbiome analysis. Their offspring (F1) were reared and developed into adulthood in chemical-free water and underwent the same assessments as F0. From these F1 fish, F2 progeny were reared, developed into adulthood in chemical-free water, and subject to the same assessments. All sample sizes are listed in Table 1.

### 2.2. Data Collection and Zebrafish Husbandry

This experiment used the 5D zebrafish line, which is a genetically diverse line [46]. Zebrafish were housed and maintained at Oregon State University’s Sinnhuber Aquatic Research Laboratory (SARL) using a published husbandry protocol (IACUC-2021-0166) [46,47]. Briefly, fish were housed in standard fish densities and fed the same as all the fish in our facility (15 fish per 2.8 L, and twice daily at 3% body weight). High quality well water was filtered for particulate matter, UV sterilized, filtered further with reverse osmosis and activated carbon, and was supplemented with Instant Ocean salts (Spectrum Brands, Blacksburg, VA, USA) and sodium bicarbonate as needed to maintain a pH of 7.4. Temperature was maintained at 28 ± 1 °C. For spawning the F1 and F2 generations, adult fish spawned at 6 months old in sex-gated tanks and embryos were collected immediately afterwards.

Morpholino and BaP exposures were performed on F0 generation embryos in concordance with previously designed protocols [48]. We chose to use a morpholino to assess AHR2 function as opposed to a genetic knockdown because we wanted the effect of AHR2 disfunction to affect only F0 fish at the exact moment we exposed to BaP, and not over the course of the lifetime or subsequent generations as a genetic line would have. We used an A-translation blocking morpholino (5′ TGTACCGATACCCGCCGACATGGTT 3′) and a standard control morpholino (5′ CCTCTTACCTCAGTTACAATTTATA 3′) purchased from Gene Tools (Philomath, OR, USA), as in previous studies [49,50]. One of these two morpholinos (~2 nL of constituted morpholino sequence) was injected into the yolk of 1–2 cell stage embryos. Phenol red dye was used to confirm successful injection. Embryos were kept in E2 embryo medium (EM) consisting of 15 mM NaCl, 0.5 mM KCl, 1 mM CaCl_2_, 1 mM MgSO_4_, 0.15 mM KH_2_PO_4_, 0.05 mM Na_2_HPO_4_, and 0.7 mM NaHCO_3_ buffered with 1 M NaOH to pH 7.2 [51]. Embryos were then stored at 28 ± 1 °C in the dark until dechorionation and subsequent BaP exposure.

Embryos were dechorionated using a custom pronase-based dechorionation instrument at 4 h post-fertilization (hpf) [52] and treated with BaP. Benzo[a]pyrene 10 mM stocks were made with 100% dry dimethylsulfoxide (DMSO) (Sigma-Aldrich [Burlington, MA, USA], 96% purity). This BaP stock was water-bath sonicated for 20 min to ensure proper dissolution, then a working stock was made using EM and sonicated for 30 min. A total of 100 µL of BaP and 0.1% DMSO control stocks were pre-loaded into the wells of round-bottom 96-well Falcon plates at concentrations of 10 µM BaP (normalized to 0.1% DMSO) or a 0.1% DMSO vehicle control. Embryos, microinjected with either a control or AHR2 morpholino, were then plated into individual wells of the plate and then moved out at 120 hpf to chemical-free EM to grow into adulthood. In adulthood, zebrafish gut microbiome and behavioral data were collected, as well as subsequent spawning and sampling of F1 and F2 generations.

Adult zebrafish were screened for standard behavioral changes at each generation (F0, F1, F2) at 8–10 months of age using previously defined assays [4,53]. Adult zebrafish were put into tanks alone and a Point Gray Camera was used in conjunction to Viewpoint ZebraLab 3.3 (Lyon, Fance) software to record the distance they swam in 1-min increments over the course of 30 min. Of note, the ZebraLab software v3.3 was used to measure F0 fish and was upgraded to v5.18 for F1 and F2, and accounts for the difference in sensitivity of measurements that is apparent in the presented analysis. Within each generation, the sample size is a normal distribution for the controls, which implies the behavior is reproducible. The software detection threshold remains the same of what is considered movement (>2 cm/s). With the upgraded camera in F1 and F2, this improvement in camera quality reduced the noise in the data. Therefore, our analysis, detailed in a later subsection, focuses on finding trends between treatments within each generation. We measured four assays: (1) free swim distance (cm), a measure of distance swum when a fish is introduced into a new tank, thus representing the exploration of an environment; (2) shoaling nearest-neighbor distance (nnd) (cm), a social behavior metric measuring the distance to the nearest neighbor in a group of four fish, where one fish is tracked; (3) shoaling inter-individual distance (iid) (cm), a social behavior measure of the distance between pairs of fish in a group of four fish; (4) shoaling speed (cm/min), a social behavior metric of the speed of a focal fish in a group of four fish. Consistent with past published work, dead or morphologically deficient fish were removed from the analysis, and the first 10 and last 8 min were trimmed from the data (fish tend to behave variably or freeze at the start because they are in a new environment and acclimate to the new tank by the end of the measurement period).

Zebrafish were sampled for gut microbiome analysis in adulthood (at least 120 days post-fertilization) at each generation and treatment group. Fish were euthanized using MS-222 at 470 mg/L for 10 min as per the accepted IACUC protocol. Fish were dissected to collect whole large and small intestines, which were stored in sterile 2.0 mL centrifuge tubes in liquid nitrogen immediately after collection and stored at −80 °C until all samples were collected and ready for DNA extraction in bulk. During dissection, the sex, length, and width of individuals were collected.

### 2.3. Processing Gut Microbiome Sequences

Samples were prepared for 16S rRNA and shotgun metagenomic sequencing. We extracted DNA using the DNeasy PowerSoil Pro DNA Extraction kits (Qiagen, Hilden, Germany). The V4 region of the 16S rRNA was PCR amplified with the EMP protocol [54] and primers (515F-806R). Prior to sequencing, we checked the product on a 1% agarose gel and pooled samples, normalizing concentrations using Qubit readings (Thermo Fisher Scientific, Waltham, MA, USA). Sequencing of the 16S rRNA region and shotgun metagenomic sequencing was performed through the Center for Quantitative Life Sciences at Oregon State University on the Illumina MiSeq high-throughput sequencer (San Diego, CA, USA) for 16S sequencing and an Illumina HiSeq 2000 sequencer (San Diego, CA, USA) for shotgun metagenomic sequencing. To obtain higher sequence resolution in the metagenomic shotgun sequence dataset, the same samples were sequenced across multiple lanes, and we concatenated files from the same sample after demultiplexing. All sequences are publicly available in an NCBI sequence repository project (PRJNA1147314).

16S sequence processing was performed with a modified DaDa2 pipeline in R developed by our lab for gut microbiome analysis (https://github.com/kstagaman/sharpton-lab-dada2-pipeline version 0.3.4 [accessed 6 January 2020]) implemented in R (version 2022.12.0) [55]. Reads were trimmed to 225 bp forward and reverse 200 bp. Reads with a quality score less than 30 were thrown out. We assigned amplicon sequence variants (ASVs) with the Silva 16s database training set (version 138.1) [56]. A phylogenetic tree was built with the Mothur (version 1.47.0) workflow [57] using the NAST algorithm from FastTree2 [58]. Before diversity-based analyses, we filtered mitochondria and chloroplast sequences and samples that contained extremely low or no counts, and 16S rRNA data were rarefied to 10,000 sequences.

To process shotgun metagenomic sequences, we used the Sharpton lab metaGTXprocessing pipeline (https://github.com/kstagaman/sharpton-lab-metaGTx.processing [accessed 5 June 2020]) which is an adapted version of the BioBakery workflow implemented in R (version 4.1.2 (1 November 2021)) [55]. Briefly, the workflow proceeds as such: kneaddata (v0.10.0) removes contaminant reads that match the host (via Bowtie2) and trims sequences (via Trimmomatic); microbial taxonomy is assigned via Metaphlan (version 3); gene families and pathways are inferred using the assigned taxonomy using Humann (v3.0.0.alpha.3) [55]. This information was summarized in three tables: taxa abundance per sample, gene family abundance per sample, and pathway abundance per sample. We used all three for exploratory and supplementary analyses but focus on the pathway abundances per sample in the analyses described in the main text. Before diversity-based analyses, we removed samples that contained extremely low or no counts compared to the rest of the samples using the raw sequence count per sample frequency distribution.

### 2.4. Analysis and Statistics

We determined whether embryonic BaP exposure, AHR2 morpholino status, or their interaction were associated with alpha diversity, beta diversity, or beta dispersion of ASVs (derived from 16S marker gene sequencing) and/or putative pathways (inferred from genes detected in shotgun metagenomic sequencing). Our response variables consisted of these adult zebrafish gut microbiome sequencing samples, as well as four adult zebrafish behavior measures (free swim distance, shoaling nnd, shoaling iid, and shoaling speed). We used a Kolmogorov–Smirnov test to evaluate whether the distributions of behavioral metrics differed between control and experimental treatments. We used linear regressions to model alpha diversity and beta dispersion as the dependent variables. For beta diversity, we optimized a dbRDA constrained ordination model then used the optimized model formula from this in a PERMANOVA to determine significance. Further, we included sex as a covariate in gut microbiome related regressions. We also identified biologically relevant ASVs and putative pathways that were indicative of BaP exposure, AHR2 morpholino treatment, or both by fitting relative abundance distributions to a compound Poisson generalized linear model with a log link function (MicroViz package [version 0.10.6] with phyloseq [version 1.42.0] objects as input) [59,60].

We used R version 4.2.2 (31 October 2022) [61] and associated packages to carry out statistical analyses and graphing. All code and associated files can be found on the project github page (https://github.com/aalexiev/ZebrafishBaPIntergenerationalMB [accessed 29 July 2024]). Graphs were produced using ggplot (version 3.4.1) [62] and the companion package ggpubr (version 0.6.0) [63]. Community diversity metrics and associated statistics were carried out with the vegan package (2.6-4) [64].

Four behavior measures were analyzed first with the same approach from past zebrafish toxicology research [4,53], and secondarily using a previously used approach where the area under the curve (AUC) was used as the dependent variable in a linear model with treatment variables (BaP exposure and AHR morpholino status) [33]. Since the camera model was updated such that F0 has less sensitive measurements than F1 and F2, we modeled and graphed each generation separately and compared metrics between treatments within each generation. After removing dead fish or samples with camera detection errors, the lowest sample count was three treatments in F0 fish that had three data points of shoaling iid. Although this is quite low, each of the 3 measured shoals is an average of 4 fish (of equal sex distribution), so there are 12 fish represented. With regard to the first approach, we produced a cumulative density distribution for each metric per treatment per generation (e.g., F0 AHR2Mo-/BaP- was one distribution, thus twelve total). Within each generation, we ran a Kolmogorov–Smirnov test comparing each treatment distribution (AHR2Mo-/BaP+, AHR2Mo+/BaP-, AHR2Mo+/BaP+) to the control (AHR2Mo-/BaP-), where a significant *p*-value indicates that the distributions are different from each other (Figure S1, Table S1). This means we did not compare the two groups of BaP-exposed fish in this analysis, but we were still able to assess how BaP and/or AHR affected fish compared to no treatments in order to address our main hypotheses. Regarding the second approach, we calculated the area under the curve (AUC) for each of four behavior metrics versus timepoint, per fish, within each generation (i.e., we filter the whole dataset to represent only F0 fish, then calculate AUC per fish on a graph of free swim distance versus timepoint, then pair these data with that fish’s metadata on what treatment each came from). We then ran a linear regression model for each generation with AUC as the independent variable and BaP exposure, AHR2 morpholino, and their interaction.

We first determined if there was an effect of BaP exposure on the gut microbiome of zebrafish and whether it was dependent on the AHR2 pathway using just F0 generation 16S and shotgun metagenomic sequence data. We tested several different gut microbiome alpha- and beta diversity metrics, which are included in the supplement. We calculated these metrics for two datasets: ASVs and metabolic pathways encoded in the metagenome, derived from 16S and shotgun metagenomic sequence data, respectively. We then applied this question across generations by including sequence data from all generations in our models as well as analyzing F1 and F2 generations individually. We used two modeling approaches with these data: (1) the same modeling approach as in F0 fish applied to each subsequent generation (F1 and F2), wherein BaP exposure, AHR2 morpholino, sex, and the interaction between each of these were independent variables and alpha- and beta diversity gut microbiome metrics were each the dependent variable, and (2) using all the data (i.e., with all three generations present) and the included “generation” as an independent variable in all models, along with its interaction with BaP and AHR2 morpholino treatments. The results of both modeling approaches were congruent with each other (Tables S2 and S3). For simplicity, we present the results of the first approach in the main text.

Several alpha and beta diversity measures are reported. For alpha diversity, we report richness and Shannon diversity metrics [65] in the main text, as these are exemplar metrics that represent the overarching trends observed across our analyses, but we also evaluated Simpson metrics [66] for all treatments and generations in the initial exploration of the data (Simpson metrics gave congruent results to Shannon). Similarly, for beta diversity, we report Bray–Curtis dissimilarity [67] in the main text, but also evaluated Jaccard [68] and Sørenson [69,70] metrics, which gave the same results. For alpha diversity metrics and beta dispersion, we ran linear regression models using AIC to choose the optimal model. For beta diversity metrics, dbRDA was used to graph the ordination and ordistep to choose the optimal model to constrain upon. We then used this optimal model from the ordination as the input to a PERMANOVA model to determine the significance and area of effect of the covariates.

We wanted to visualize how beta diversity varied across the three generations within each treatment, so we created a Bray–Curtis based metric to evaluate this. We first calculated Bray–Curtis dissimilarity for the full dataset. Then, we calculated the difference in dissimilarity between the F0 centroid all points in the F0 generation (as a baseline), F1, and F2. We then normalized to the “F0 centroid to F0 points” distance so that all distances for F1 and F2 were relative to F0, and F0 was on average zero. For example, the F0 metric was calculated as follows: distance from centroid F0 to each point in F0 divided by median distance from centroid F0 to each point in F0, minus 1. The calculation for F1 was distance from centroid F0 to each point in F1 divided by median distance from centroid F0 to each point in F0, minus 1. This represents, essentially, the dissimilarity in microbiomes between each F1 and F2 compared to F0. We performed this for each exposure/morpholino treatment individually. We use this measure to compare gut microbiome beta diversity trends across generations.

Subsequently, indicator analyses were performed with the MicroViz package (version 0.10.6) [59], using a compound Poisson generalized linear model to fit each taxon or pathway abundance distribution to BaP exposure, AHR2 morpholino status, or a combination of both. We used the incidence-rate ratio (IRR) to represent the degree and direction of interaction and graphed the log(IRR) for ease of interpretation (i.e., a positive value is an increased ASV/pathway relative abundance associated with that variable). Significance was determined via Wald test and *p*-values were FDR-adjusted (alpha = 0.05).

## 3. Results

### 3.1. Transient Suppression of AHR2 During Development Results in Anxiety and Altered Social Behavior

**Figure 2:**
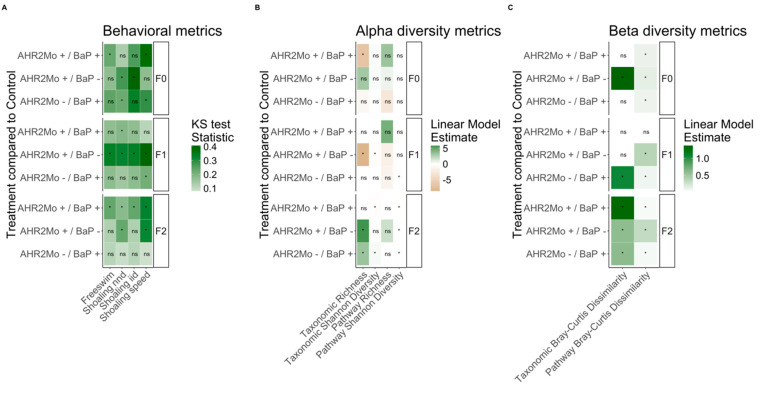
Summary heat map of statistical outcomes from behavioral and gut microbiome analyses where experimental treatments were compared relative to the control (AHR2Mo-/BaP-). (**A**) The behavioral metrics and how they associate with each experimental treatment, per generation. The KS statistic was used and is represented by the color gradient. (**B**) The gut microbiome alpha diversity metrics (based on 16S [taxonomic] and shotgun metagenomic [pathway] data) associated with the treatments, per generation. (**C**) The gut microbiome beta diversity metrics (again, for 16S and shotgun metagenomic data) associated with the treatments, per generation. In Panels B and C, the color gradient represents the linear model estimate (i.e., the slope of the association). Ns means not significant, and the asterisk represents significant associations. Tables S2 and S3 (rows labeled “F0”) show expanded model results and Table 1 has sample sizes.

**Figure 3:**
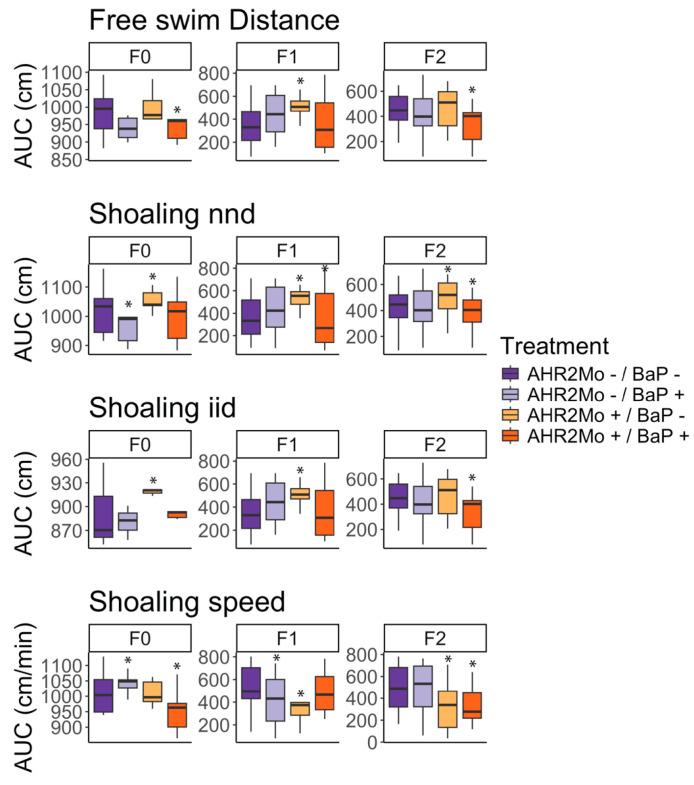
Box and whisker plot of the differences in zebrafish behavior across experimental groups and generations. Four behavior metrics (free swim distance, shoaling nnd, shoaling iid, and shoaling speed) were measured using a ZebraBox camera, then used to create a cumulative density function (CDF) distribution for each treatment, per generation. CDF distributions were evaluated via a Kolmogorov–Smirnov (KS) test comparing each treatment CDF distribution to that of the control (AHR2Mo-/BaP-), for each generation, per metric. Here, we present box and whisker plots of the area under the curve (AUC) of the CDF distribution for each treatment, organized by generation and metric, to portray trends from the KS test visually. The asterisks indicate significance (*p* ≤ 0.05) in experimental treatments relative to the control (AHR2Mo-/BaP-, dark purple), derived from the KS test described above (Table S1). Each color indicates the treatment. The center line of each box represents the median, the whiskers of each box are the standard deviation. Note that the y-axes for each plot show different scales, in order to more easily visualize trends within each data subset.

Previous studies assessing the impacts of the developmental exposure of BaP on anxiety and social behaviors in adult zebrafish showed that the changes in behavior were heritable [4]. In this study, we evaluated social perception using adult shoaling behavior metrics—inter-individual distance (iid) (cm), nearest neighbor distance (nnd) (cm), and shoaling speed (cm/min)—and evaluated anxiety using the free swim distance (cm) of adult fish when introduced into a new tank (Figure 2A, Figure 3 and Figure S1; Table S1). Taken together, perturbations in any of these metrics relative to the control group (AHR2Mo-/BaP-) would indicate that behavior is altered.

F0 fish showed several disturbed behavior metrics due to AHR2Mo+ treatment. We found that the two treatment groups not exposed to BaP (AHR2Mo-/BaP- and AHR2Mo+/BaP-) exhibited statistically different social behaviors based on AHR2Mo treatment. The fish with a transient knockdown of AHR2 during development had less variation than control fish (Figure 2A and Figure 3, Table S1). These AHR2Mo+ fish exhibited increased median iid (KS test: *p* = 0.02) and nnd (KS test: *p* = 0.004), which indicates a less cohesive shoal, and this trend persisted in F1 and F2 fish (Figure 2A and Figure 3, Table S1). F1 and F2 fish additionally exhibited slower shoaling speeds (KS test: *p* = 0.01 and 0.00001, respectively) (Figure 2A and Figure 3, Table S1). Hypoactive swimming and the desire to be in a less cohesive shoal indicates that the AHR2Mo treatment is associated with anxiety-like behavior that was heritable across generations.

When considering BaP’s impact on neurobehavior across a generation independent of AHR2 (AHR2Mo-/BaP+), the results were consistent with our previous study that demonstrated a change in social behaviors in F0 fish (KS test: decreased nnd; *p* = 0.03; higher shoaling speed; *p* = 0.02) (Figure 2A and Figure 3, Table S1) [4]. The F1 generation did not show as robust a social perception change as F0, with the only statistically significant changes observed relating to a decrease in shoaling speed (KS test: *p* = 0.02) (Figure 2A and Figure 3, Table S1). By F2, the abnormal social behavior was not detected at all in AHR2Mo-/BaP+ fish.

Overall, BaP and AHR2 morpholino treatment perturbed movement and social cohesiveness in zebrafish, and these effects manifested over generations. The role of AHR2 in BaP neurotoxicity was evaluated by comparing the AHR2Mo+/BaP+ treatment group and the control (AHR2Mo-/BaP-). There was an impact on the F0 social cohesion (KS test: slower shoaling speed; *p* = 0.00004) and hypoactive swimming (KS test: *p* = 0.05) (Figure 2A and Figure 3, Table S1). F1 and F2 exhibited disrupted social behaviors and swimming behaviors as well. F1 fish exhibited a slightly lower median nnd with more variation (KS test: *p* = 0.05) but no change in swimming behavior (Figure 2A and Figure 3, Table S1). By F2, the fish exhibited hypoactive swimming and decreased social cohesion (KS test: free swim, *p* = 0.004; nnd, *p* = 0.05; iid, *p* = 0.004; speed, *p* = 0.000009) (Figure 2A and Figure 3, Table S1). Collectively, F0 and F2 fish in the AHR2Mo+/BaP+ treatment exhibited hypoactive swimming and decreased social cohesion, which are markers of anxiety-like behavior.

### 3.2. Developmental BaP Exposure, AHR2 Disruption, and Their Interaction Affect Gut Microbiome Metrics in F0 Fish

To determine if the microbiome is linked to BaP and AHR2 morpholino exposure, we first evaluate how the microbiome differs across experimental groups in fish directly exposed to these conditions (i.e., F0 fish). First, we found that BaP exposure elicits modest effects on the gut microbiome. Neither taxonomic alpha diversity nor beta diversity was associated with BaP exposure as a main effect (Figure 2B,C). However, BaP exposure was associated with bacterial metabolic pathway beta diversity (PERMANOVA: R^2^ = 0.06, *p* = 0.001), and metabolic pathway richness approached significance (linear repression: coefficient = −2.8, *p* = 0.09). Beta dispersion was not associated with any experimental variables in taxonomic or pathway gut microbiome datasets (Tables S2 and S3).

**Figure 4:**
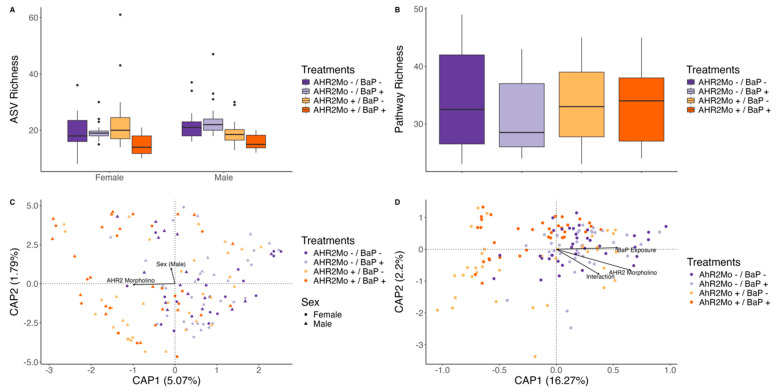
Plots showing alpha and beta diversity metrics in taxonomic and pathway data per significant treatments (identified via regression approaches) for generation F0. (**A**) A box and whisker plot showing the ASV richness for each treatment (color) per sex (linear regression: exposure and morpholino interaction, coefficient = −6.8, *p* = 0.002; morpholino and sex interaction, coefficient = −5.3, *p* = 8.3 × 10^−5^). The center line is the median and the whiskers show the standard deviation. (**B**) A box and whisker plot showing pathway richness for each treatment (color) (linear regression: no significant differences). The center line is the median and the whiskers show the standard deviation. (**C**) A dbRDA ordination showing taxonomic beta diversity (Bray–Curtis dissimilarity) per treatment (color) and sex (shape) (PERMANOVA: *p* = 0.001 and 0.03, respectively). (**D**) A dbRDA ordination showing the pathway beta diversity (Bray–Curtis dissimilarity) per treatment (color) (PERMANOVA: *p* = 0.001 for all three covariates on the vectors).

That said, BaP elicits strong effects on the gut microbiome in an AHR2-dependent manner. For example, the interaction between AHR2 morpholino treatment and BaP exposure was correlated with ASV richness (linear regression: coefficient = −6.8, *p* = 0.002), whereas each individual variable (AHR2 morpholino and BaP exposure) was not significant (Figure 2B, Table S2). The control cohort (AHR2Mo-/BaP-) had the highest ASV richness, whereas AHR2Mo+/BaP+ fish had the lowest (Figure 4A). In addition, the interaction between BaP exposure and AHR2 morpholino was also associated with bacterial pathway beta diversity (PERMANOVA: R^2^ = 0.07, *p* = 0.001) (Figure 2C, Figure 4C and Figure S2, Table S3). There was no effect of the interaction on beta dispersion (Tables S2 and S3). Taken together, these results point to an AHR2-dependent BaP exposure effect on the gut microbiome.

In addition to influencing the effect of BaP on the microbiome, we found that AHR2 morpholino treatment elicited an effect on the zebrafish gut microbiome independent of BaP exposure. In fact, the effect of AHR2 morpholino treatment on the gut microbiome was stronger in many cases than that of BaP exposure (Figure 2B,C and Figure 4C,D). ASV richness and Shannon diversity were each associated with morpholino treatment; however, this association differed as a function of sex (linear regression: richness, coefficient = −5.3, *p* = 0.01; Shannon diversity, coefficient = −0.5, *p* = 8.3 × 10^−5^) (Figure 2B, Figure 4A and Figure S2). For example, ASV Shannon diversity in female AHR2Mo+ fish was higher than that of female AHR2Mo- zebrafish, whereas this pattern was reversed in males (Figure 2B and Figure S3). Moreover, ASV Bray–Curtis dissimilarity was associated with AHR2 morpholino treatment (PERMANOVA: R^2^ = 9.3, *p* = 0.001) (Figure 2C and Figure 4B). Pathway Bray–Curtis dissimilarity was also associated with morpholino treatment (PERMANOVA: R^2^ = 16.2, *p* = 0.001) (Figure 2C), as was beta dispersion, albeit more weakly (linear regression: coefficient = 0.01, *p* = 0.052) (Figure S2).

**Figure 5:**
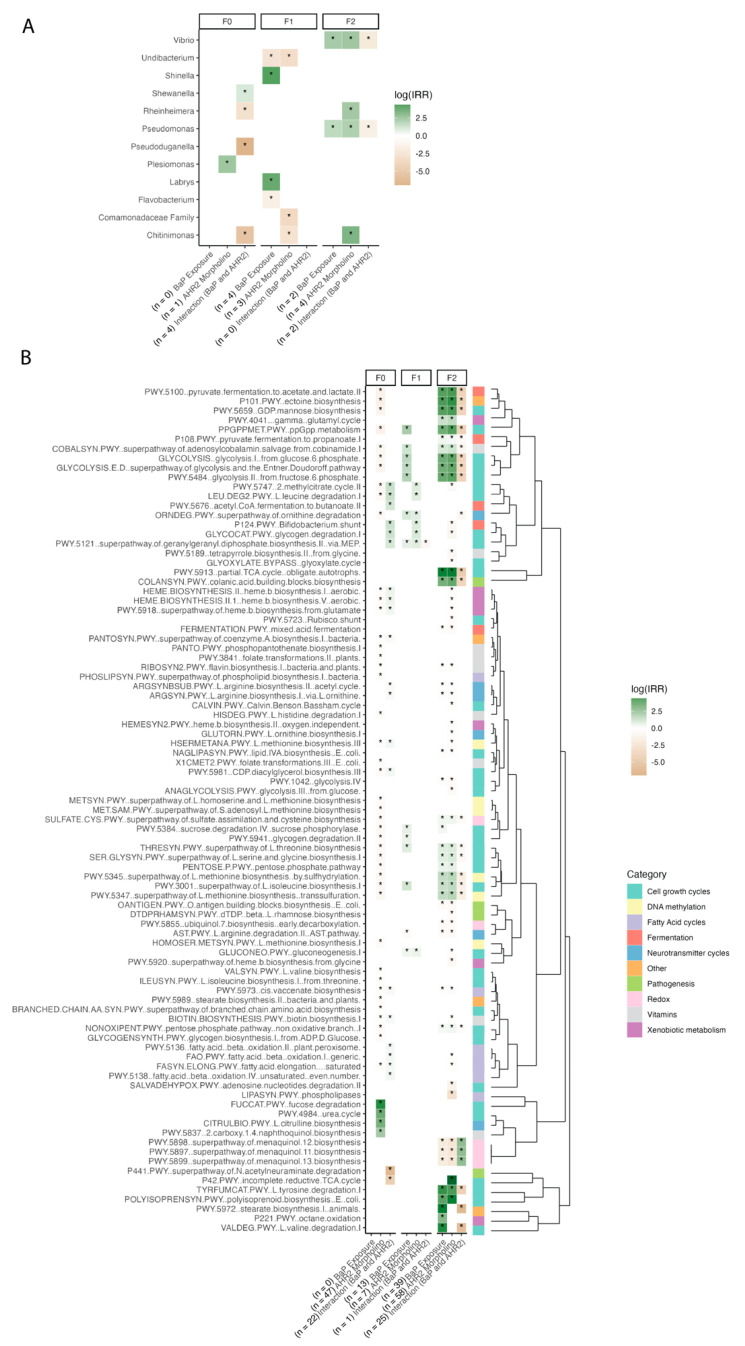
A heatmap of indicator taxa and pathways associated with model terms, per generation, with number (n) of significant associations in parentheses by each model term. (**A**) This shows indicator taxa listed on the y-axis while (**B**) shows the indicator pathways, organized by a dendrogram based on the Bray–Curtis matrix (right), and each organized by generation (top labels). Each y-axis lists the model terms used in a negative binomial model of each taxon/pathway relative abundance, along with the number (n) of significantly associated taxa (**A**) or gene pathways (**B**) shown in parentheses by the corresponding model term. Log(IRR) is represented by a color scale, which is the strength and direction of the association wherein positive numbers are dark green and show a positive association between the taxon/pathway relative abundance and the model term, and vice versa for negative numbers. The bottom set of colors in panel B indicates the broad category each pathway falls under (curated using available information from BioCyc and literature searches). Asterisks represent significant *p*-values from the model.

Supporting the above findings, several ASVs and microbial gene pathways in the zebrafish gut were correlated with AHR2 morpholino status and the interaction between BaP exposure and AHR2 morpholino status (but not BaP exposure alone) (Figure 5, F0 labels in heatmap). One example is *Shewanella*, a member of the core zebrafish microbiome [16,71], whose relative abundance increased in association with developmental BaP exposure in an AHR2-dependent manner (Wald test: interaction between exposure and morpholino, *p*-value ≤ 0.05) (Figure 5A). Additionally, *Rheinheimera*, *Pseudoduganella*, and *Chitinimonas* relative abundances decreased in association with morpholino and exposure treatment together (Wald test: *p*-value ≤ 0.05), while *Plesiomonas* relative abundance increased with morpholino treatment (Wald test: *p*-value ≤ 0.05) (Figure 5A). We also detected bacterial pathways related to xenobiotic and neurotransmitter (e.g., Gamma-aminobutyric acid [GABA]) metabolism, DNA methylation, fermentation, short chain fatty acid synthesis and degradation, and cell growth (TCA cycle and cell membrane metabolism especially) that are enriched or depleted in association with AHR2 morpholino treatment and its interaction with BaP exposure, but not exposure alone (Figure 5B, Table S4). This is represented in the number of significant associations listed next to each variable on the x-axis. These results further support the finding that specific ASVs and pathways in the gut microbiome respond to BaP exposure in an AHR2-dependent manner and AHR2 itself has a strong effect on gut microbiome succession from embryo to adulthood.

### 3.3. Gut Microbiome and Behavior Changes Associated with BaP Exposure and AHR2 Morpholino Persist over Subsequent Generations

We found that overall, patterns of association between experimental factors and gut microbiome metrics persisted in subsequent generations. Specifically, we found that BaP exposure, AHR2 morpholino status, and the interaction between the two had a strong association with alpha- and beta diversity metrics in subsequent generations. In some cases, these effects were stronger than those observed in F0 fish.

**Figure 6:**
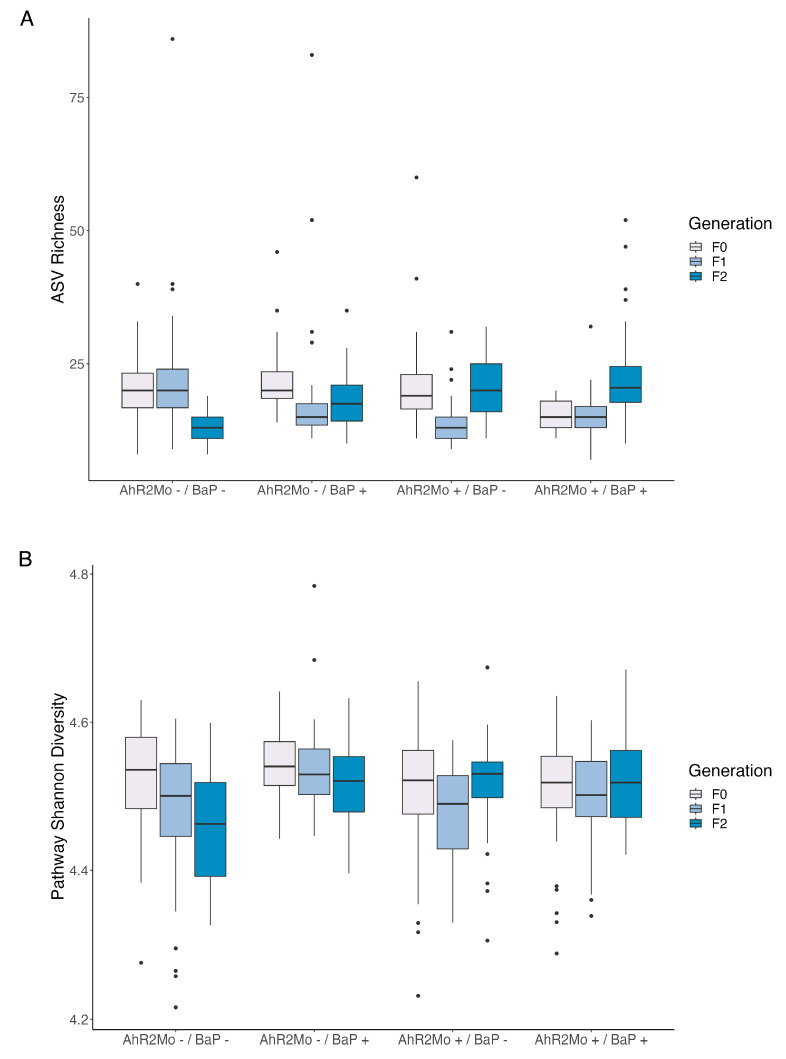
Box and whisker plots of alpha diversity metrics per treatment in each generation, for taxonomic and pathway diversity. (**A**) Taxonomic richness and (**B**) pathway Shannon index were the only significant metrics (linear regression: *p* ≤ 0.05) among all alpha diversity metrics tested (see Table S1 for statistics). Color in both graphs shows generations F0–F2 and x-axis lists the different treatments. The center line is the median and the whiskers show the standard deviation.

Linear regression models showed that BaP exposure and AHR2 morpholino treatments in F0 embryos are overall strongly associated with the alpha diversity of gut microbiomes in F1 and F2 fish. In F1 fish, ASV richness and Shannon diversity were both associated with AHR2 morpholino treatment (richness coefficient = −8.6, Shannon diversity coefficient = −0.2, *p* = 0.005 for both metrics), but not exposure (Figure 2B and Figure 6A; Table S2). By contrast, metabolic pathway Shannon diversity was associated only with BaP exposure (coefficient = 0.07, *p* = 0.0005) (Figure 2B and Figure 6B; Table S2). In F2 fish, ASV alpha diversity was associated with morpholino status (richness, coefficient = 5.8, *p* = 1.2 × 10^−7^), exposure (richness coefficient = 3.2, Shannon diversity coefficient = 0.3, *p* = 0.002 for both), and their interaction (Shannon diversity, coefficient = −0.2, *p* = 0.05) (Figure 2B and Figure 6A; Table S2). Pathway Shannon diversity of F2 fish microbiomes was associated with BaP exposure (coefficient = 0.06, *p* = 0.0002), morpholino status (coefficient = 0.06, *p* = 0.0002), and their interaction (coefficient = −0.06, *p* = 0.01) (Figure 2B and Figure 6B; Table S2).

**Figure 7:**
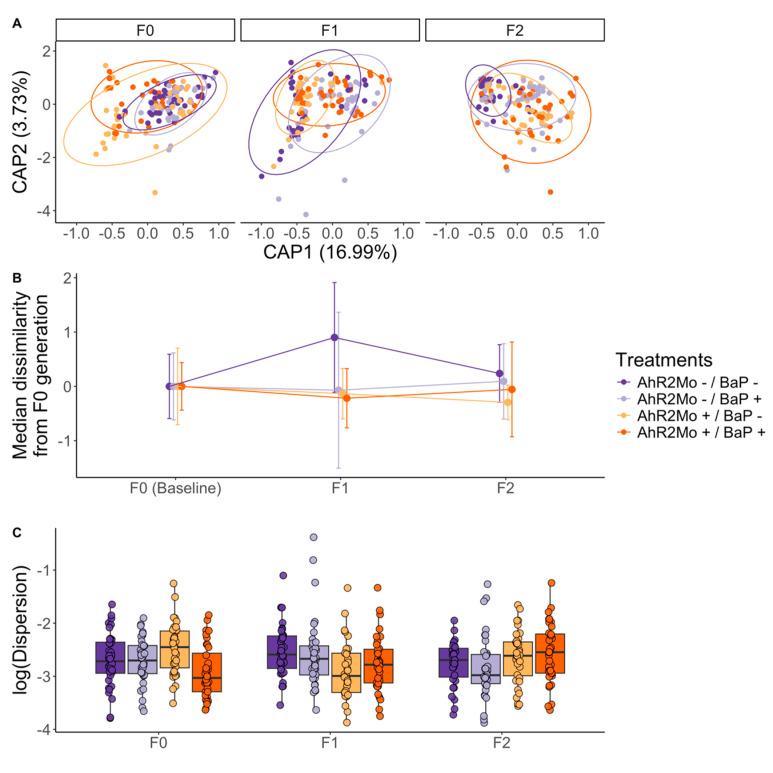
Bray–Curtis dissimilarity of functional pathways across generations and treatments across the whole dataset. (**A**) Represents a dbRDA ordination where each point is a zebrafish gut microbiome sample, with color showing treatments and the ordination is faceted by generation. The ellipses show each generation per treatment grouping (n = 12) where color is treatment. (**B**) A dot plot of the median distance from the points in each generation–treatment combination to the F0 centroid for the corresponding treatment (see methods for details). Distances are taken from the dbRDA ordination in Panel A, normalized to the F0 centroid, and parsed by generation and treatment (color). Lines colored by treatment connect each treatment across generations. The vertical brackets show the standard deviation on each dot in the plot. (**C**) A box and whisker plot of the dispersion values for each treatment (colors) within each generation. Dispersion values have been log transformed for ease of visualization. The center line is the median and the whiskers show the standard deviation. The legend is common to all three panels.

PERMANOVA models of beta diversity also support the finding that BaP and AHR2 perturbations have sustained effects on gut microbiome composition and functional capacity in subsequent generations. Pathway Bray–Curtis dissimilarity was associated with morpholino status (R^2^ = 0.17, *p* = 0.001) and BaP exposure (R^2^ = 0.03, *p* = 0.002) (Figure 2C, Figure 7A and Figure S3B; Table S3). Further, we observed that all treatments deviated from the control in terms of median Bray–Curtis dissimilarity in F1 and F2 generations relative to F0 (Figure 7B; Table S3). F1 was particularly dissimilar from F0, whereas F2, despite being further removed from F0, was more similar to F0 than F1 was, congruent with a transgenerational effect of BaP and AHR2 on gut microbiome beta diversity (Figure 7B; Table S3). Beta dispersion was associated with BaP exposure and AHR2 morpholino treatment in the F1 generation only (linear regression; BaP coefficient = 0.03, *p*-value = 0.04; AHR2 coefficient = −0.03, *p*-value = 0.02) (Figure 7C; Table S3). ASV Bray–Curtis dissimilarity in F1 fish was associated with BaP exposure (R^2^ = 0.04, *p* = 0.001) (Figure S3A; Table S3). In F2 fish, pathway Bray–Curtis dissimilarity in F2 fish was also associated with morpholino status (R^2^ = 0.21, *p* = 0.001), BaP exposure (R^2^ = 0.03, *p* = 0.002), and their interaction (R^2^ = 0.02, *p* = 0.004) (Figure S3B), and the beta dispersion of pathways was associated with morpholino status (linear regression: coefficient = 0.02, *p* = 0.01) and BaP exposure (linear regression: coefficient = 0.02, *p* = 0.02) (Table S3). ASV Bray–Curtis dissimilarity was associated with morpholino status (R^2^ = 0.06, *p* = 0.001), BaP exposure (R^2^ = 0.03, *p* = 0.005), and their interaction (R^2^ = 0.03, *p* = 0.02). The beta dispersion of ASVs in F2 fish was associated with morpholino status (linear regression: coefficient = 0.09, *p* = 0.005) (Table S3).

Several ASVs and metabolic pathways were associated with BaP exposure, AHR2 morpholino status, and their interaction within each generation (Figure 5). For example, the Comamonadaceae family, a core zebrafish group [16], exhibited decreased relative abundance with AHR2 morpholino treatment in generation F1 (Wald test: *p*-value ≤ 0.05) (Figure 5A). In the F2 generation, ASVs from the core zebrafish gut genera *Vibrio* and *Pseudomonas* [16] both exhibited increased relative abundance when exposed to BaP and the AHR2 morpholino (Wald test: *p*-value ≤ 0.05), and had decreased relative abundance in association with treatment with both BaP and AHR2 morpholino (*p*-value ≤ 0.05) (Figure 5A). Further, there were several pathways that were associated with BaP and/or AHR2 morpholino to various extents (Figure 5B), again including pathways linked to xenobiotic metabolism, neurotransmitter metabolism, DNA methylation, fermentation, short chain fatty acid metabolism, and cell growth. Extended explanations of each pathway as well as the superclasses from BioCyc can be found in Table S3. Broadly, the associations were stronger and increased in the F2 generation (and weak or not at all present in the F1 generation by comparison, and less so in F0) or were maintained between subsequent generations, as is shown in the enumerated differentially abundant pathways listed in the x-axis of Figure 5B. This observation is consistent with the alpha- and beta diversity results pointing to the intergenerational effects of BaP exposure and AHR2 perturbation on the zebrafish gut microbiota, spanning differences in taxonomic composition as well as functional capacity (Figure 5B).

## 4. Discussion

Our investigation sought to address three key questions: (1) how exposure to BaP and developmental expression of AHR2, a key gene in the zebrafish AHR pathway, links to adult zebrafish behavior; (2) how these same variables associate with the structure and function of the adult zebrafish gut metagenome; and (3) whether these associations subsequently manifest across three generations of fish. We found that exposure to BaP had a modest effect on zebrafish behavior and gut microbiome, but this effect was stronger when the AHR2 gene was perturbed, implying that BaP affects zebrafish behavior and the gut microbiome (composition, structure, and functional capacity) in an AHR2-dependent manner. These associations also persisted and, in some cases, strengthened over subsequent generations (F1 and/or F2). Lastly, we found that AHR2 had a strong effect on zebrafish behavior and gut microbiome assembly independently of BaP exposure across all generations. Overall, these results support the hypothesis that BaP perturbs the successional development of the gut microbiome to dysregulate behavior in an AHR2-dependent manner. Below, we expand on the major patterns we observed in the data and their implications, given prior research.

Embryonic BaP exposure elicits modest effects on zebrafish gut microbiome and behavior in adult zebrafish. In our analysis of fish directly exposed to BaP (i.e., F0 AHR2Mo- zebrafish), we found that BaP elicits significant effects on two measures of adult fish behavior: shoaling nnd and shoaling speed (Figure 2A). The most directly comparable past study we know of uncovered a stronger BaP effect on specific behavior measures, like shoaling nnd and iid, but ultimately found the same outcome—that social behavior is perturbed by BaP [4]. This slight difference could also stem from updated methodology in the behavior assays. Beta diversity of the gut microbiome’s functional capacity in F0 fish shifted with BaP exposure as well (Figure 2C). This result indicates that, like behavior, the successional dynamics of the gut microbiome over the course of fish development are modestly impacted by embryonic BaP exposure. Past research also measured a modest effect of embryonic BaP exposure at increasing doses on larval zebrafish gut microbiome structure and composition [33]. Another study found similarly modest shifts in gut microbiota and moderate inflammation of the gut epithelium in mice that were dosed with BaP in adulthood and then promptly measured [72]. However, this study differed from ours in that they did not measure the effect of developmental BaP exposure on the adult gut microbiome or behavior.

In contrast to the modest effects described above, exposing embryos to both BaP and the AHR2 morpholino results in a strong effect on both behavior and the gut microbiome. As far as we are aware, no study has previously tested the effect of early life AHR perturbation alongside developmental BaP exposure on the gut microbiome. In many instances, we found that the effect of BaP on the gut microbiome is sensitive to the state of AHR2 functioning at the time of exposure (Figure 2). For instance, a large number of bacterial pathways involved in xenobiotic response and gut–brain crosstalk were not differentially abundant in association with BaP exposure unless AHR2 was also disrupted (Figure 5B). This result indicates that the AHR2 expression state at the time of BaP exposure dictates how the host and its microbiome respond to BaP exposure. The importance of AHR2 functioning makes sense because it binds numerous environmental chemicals, not only BaP, in addition to microbially derived metabolites [3,15,39,42,73,74,75]. Additionally, our prior work found that developmental BaP exposure elicits a modest effect on gut microbiome assembly in larval zebrafish [33]. BaP binds to the AHR2, resulting in DNA adducts that are sometimes not cleared and themselves are related to neurotoxicity [5], so it is possible that the reason we see an interaction effect of BaP and AHR2 on the microbiome is attributable to the variation in these BaP catabolites, which may perturb the microbial community.

Our study also uncovered evidence that developmental BaP exposure can elicit multigenerational effects on the gut microbiome and behavior, and that these effects are mediated alongside developmental AHR2 expression state. For example, BaP exposure exhibits stronger effects on behavior and gut microbiome metrics in F1 and F2 fish compared to F0 fish. Specifically, BaP exhibits an inter- and transgenerational effect on gut microbiome alpha- and beta diversity metrics, many taxonomic and pathway bacterial relative abundances, and a variety of behavior measures. These multigenerational effects were even larger in fish subject to AHR2Mo+ injection, indicating that AHR2 may influence how BaP manifests its effects across generations.

This is the first study to demonstrate that early life exposure to BaP has a generational behavior effect that is dependent on AHR2 in zebrafish wherein these effects are accompanied by perturbations to the gut microbiome. Specifically, transgenerational effects (i.e., F1 shows a modest response compared to F0 and F2), such as those observed here, imply that an epigenetic mechanism may be at play [76,77]. We found that bacterial pathways related to methylation cycles were differentially abundant in association with BaP and/or AHR2 perturbation within each generation (Figure 5). Past research also found that BaP metabolism by AHR leads to the production of DNA adducts and alterations to the DNA methylome of vertebrates [2,4,5,12,48,78,79]. BaP also induces multigenerational behavioral effects in association with changes in zebrafish genome-wide DNA methylation [3,13]. This is in line with past studies that have found transgenerational patterns in behavioral perturbations following toxicant exposures, which is indicative of an epigenetic mechanism [4,76,77]. Notably, both gut microbiome and behavior manifested transgenerational effects of AHR2 perturbation in the present study. This finding supports the idea that epigenetics are at play to some extent and identifies the gut microbiome as an additional target of epigenetic modification beyond just vertebrate DNA. Supporting this hypothesis, prior work in mouse studies found that changes in the epigenetic state of the hippocampus was linked to alterations in the gut microbiome [80]. Future work should clarify the role of exposure-induced variation on the epigenome of zebrafish and the gut microbiome, and the consequences on the microbiome, host behavior, and the gut–brain axis.

Additionally, developmental AHR2 state is associated with the behavior and gut microbiome in adult zebrafish and their progeny. We found that developmental AHR2 morpholino treatment perturbs adult behavior and gut microbiome assembly, sometimes more strongly than BaP exposure, and this effect spanned all three generations (Figure 2). AHR regulates gut homeostasis by binding bacterially produced metabolites of tryptophan, mainly indoles, kynurenine, and serotonin [19,39,42], and AHR2 function in zebrafish is required for normal behavioral development [81]. Early-life exposures to other AHR agonists had a similar effect and caused gut microbiome dysbiosis [22,82]. AHR ligands like tryptophan and indole compounds have also been associated with neurodegenerative and neurological disorders and central nervous system inflammation [19,42,83]. Consistent with this previous research, we identified various bacterially derived neuromodulator pathways that were differentially abundant in association with AHR2 morpholino injection, including tryptophan (a serotonin precursor), short-chain fatty acids, and GABA (a neurotransmitter) [19,42] (Figure 5B). One specific example is the L-Ornithine biosynthesis pathway (Table S3), which has slightly decreased abundance in F2 fish derived from F0 fish that had AHR2 morpholino treatment (Figure 5B). L-Orthinine biosynthesis increases the level of AHR ligand L-kynurenine, produced from tryptophan metabolism in gut epithelial cells [84]. Additionally, one review hypothesized that BaP binds to AHR to then transcribe a N-methyl-d-aspartate glutamate receptor (NMDAR), which in turn, mediates neurotoxic effects [7]. To do this, NMDAR binds glutamate, and we identified differentially abundant pathways related to glutamate metabolism cycles in association with AHR2 (Figure 5B). These various lines of evidence together suggest that perturbing AHR2 function for a temporary moment in early life has some effect on the functional capacity of gut microbiota. These metabolites are not only important for behavioral development and establishing homeostasis between the gut and brain, but also aid in the further assembly of the adult gut microbiome. The results underscore the importance of AHR2 to zebrafish development, particularly in the context of the gut–brain axis, and future work is needed to clarify the mechanisms through which AHR2 drives neurodevelopment.

Taken together, observations stemming from this study support the hypothesis that BaP exposure interacts with AHR2 to elicit neurotoxicity and dysregulate gut microbiome assembly and the gut–brain interaction. While our work suggests that BAP interacts with AHR2 and the gut microbiome in ways that associate with neurotoxicity in zebrafish, it will be important for further studies to evaluate the translational context of our research. Zebrafish harbor an expanded set of homologs of the AHR and CYP genes found in humans [36,85]; therefore, the routes and sensitivity of exposure may differ between zebrafish and humans. The AHR2 gene in zebrafish is orthologous to the human AHR responsible for binding BaP [36]. Despite differences, zebrafish also share similar physiological and xenobiotic pathways to humans and other mammals and have been a well-established model for developmental toxicology [86,87]. This study also raises the hypothesis that environmental chemical exposure may elicit multigenerational dysbioses in the gut microbiome. Future work should characterize the mechanisms behind how different gut microbiota interact with BaP in the gut, whether BaP and microbial ligands somehow interact to bind to AHR2 in the context of zebrafish neurotoxicity, and how these effects propagate to subsequent generations. Future work should also consider toxicants that bind AHR2, like other polycyclic aromatic hydrocarbons, 2,3,7,8-Tetrachlorodibenzodioxin, and polychlorinated biphenyls, to determine if the interactions identified here manifest in other chemicals. Finally, our study highlights the utility of the zebrafish model in these efforts, as it offers tools to explore how host genetics interact with the gut microbiome to clarify neurotoxicity mechanisms, scalability to resolve otherwise subtle, but biologically important, effects, and rapid growth to afford insight into multigenerational effects.

## 5. Conclusions

The mechanisms behind BaP’s neurotoxicity are complex and difficult to pinpoint. We found that BaP affects the gut microbiome alongside behavior in an AHR2-dependent way. This effect is generational in such a way that suggests an epigenetic mechanism, something that should be further investigated in future studies. AHR2 disruption had a distinct effect on both gut microbiome metrics and behavior, suggesting that AHR2 could somehow modulate the gut–brain axis, presenting another important area of further investigation. The present study supports the notion that integrating the gut microbiome as a response variable in toxicant studies beyond just BaP is an important shift in the field of toxicology, especially considering that AHR binds a broad spectrum of toxicants and microbial metabolites.

## Figures and Tables

**Table 1:** Sample sizes for microbiome and behavioral statistical analyses throughout. In stool samples and free swim, count refers to the number of individual zebrafish in each statistically relevant category listed in the rows of the chart. The unbolded categories are sample numbers within the bolded ones above them. For all shoaling behaviors, count refers to the number of shoals (four fish each) in each statistically relevant category. The sample sizes listed are calculated after dropping low-quality samples for each type of dataset (see methods for details on criteria). These sample sizes represent biological replicates.

	Count of Stool Samples	Count of Free Swim Samples	Count of Shoaling iid Samples	Count of Shoaling nnd Samples	Count of Shoaling Speed Samples
**F0**	**144**	**31**	**15**	**42**	**32**
AhR2Mo+/BaP+	36	10	6	12	11
AhR2Mo+/BaP-	36	9	3	12	9
AhR2Mo-/BaP+	36	6	3	9	6
AhR2Mo-/BaP-	36	6	3	9	6
**F1**	**139**	**42**	**42**	**42**	**42**
AhR2Mo+/BaP+	36	10	10	10	10
AhR2Mo+/BaP-	33	8	8	8	8
AhR2Mo-/BaP+	36	12	12	12	12
AhR2Mo-/BaP-	34	12	12	12	12
**F2**	**139**	**54**	**54**	**54**	**54**
AhR2Mo+/BaP+	36	13	13	13	13
AhR2Mo+/BaP-	36	14	14	14	14
AhR2Mo-/BaP+	36	14	14	14	14
AhR2Mo-/BaP-	31	13	13	13	13
**Grand Total**	**422**	**127**	**111**	**138**	**128**

## Data Availability

All sequences are publicly available in an NCBI sequence repository project (PRJNA1147314). All code and associated files can be found on the project github page (https://github.com/aalexiev/ZebrafishBaPIntergenerationalMB [accessed 29 July 2024]).

## Supplementary Materials

The following supporting information can be downloaded at: https://www.mdpi.com/article/10.3390/toxics13010010/s1. Figure S1. CDF distributions of four behavioral measures. Each of four behavior measures are shown per row, while the generations are shown per column. Each color represents one of four treatments; the KS test was run between the control treatment (AHR2Mo-/BaP-, the dark purple line) and the other treatments. See Figure 7 and Table S4 for which statistical comparisons were significant. Figure S2. Box and whisker plot of taxonomic Shannon diversity. The plot is facet wrapped by sex of the fish and the morpholino type is shown along the x-axis. Sex (F for female, M for male) and morpholino status (AHR2Mo representing AHR2 morpholino treatment and CoMo representing the control morpholino) were the two covariates retained in the optimal linear regression model. Figure S3. (A) dbRDA ordinations showing the differences between each generation of zebrafish using the 16S data. Each plot is labeled at the top with the corresponding generation. The colors show treatments and shapes show sex. (B) Likewise, the shotgun metagenomic data dbRDA ordinations per generation and the colors show treatments. Table S1. Statistical outcomes from behavioral analysis, using the KS test to compare the CDF of the control group (AHR2Mo-/BaP-) to each treatment group. Table S2. All taxonomic (16S) models used to test whether microbial taxonomic diversity metrics were associated with different experimental treatment variables. Table S3. All pathway (metagenomic) models used to test whether microbially derived pathway diversity metrics were associated with different experimental treatment variables. Table S4. A table of the significantly associated pathways, noted using BioCyc ID, Figure 5B, their superclasses from BioCyc, and the information we were able to find about them through BioCyc and a literature search.
