# Supplementary figures and images for: Clearing the Air on Pollutant Disruptions of the Gut–Brain Axis: Developmental Exposure to Benzo[a]pyrene Disturbs Zebrafish Behavior and the Gut Microbiome in Adults and Subsequent Generations
Source: Toxics. 2024 Dec 25;13(1):10. doi: 10.3390/toxics13010010 (PMC11768907; doi:10.3390/toxics13010010)

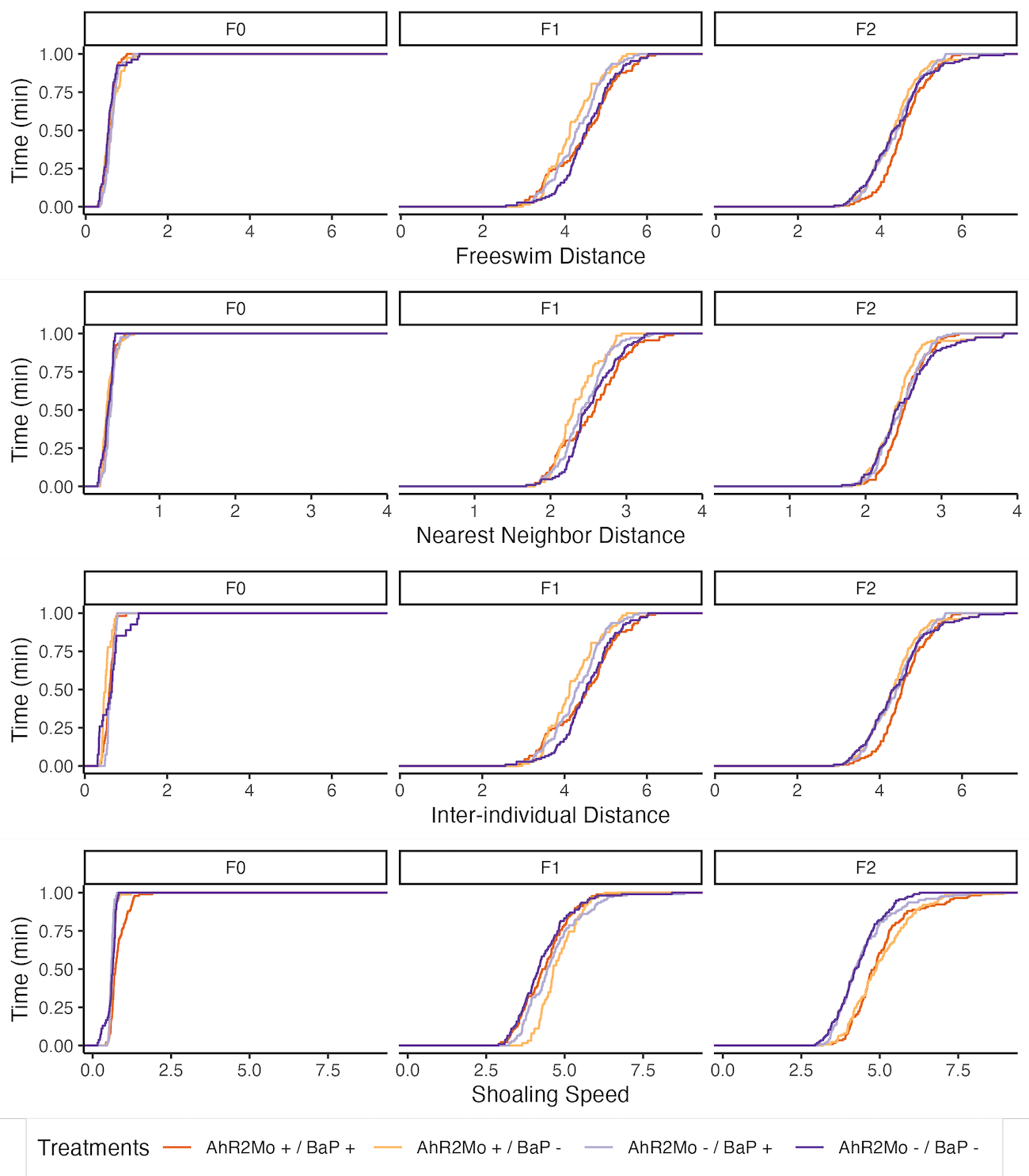

Supplement: Supplementary file 1 [file toxics-13-00010-s001.zip › FigS1.tiff]

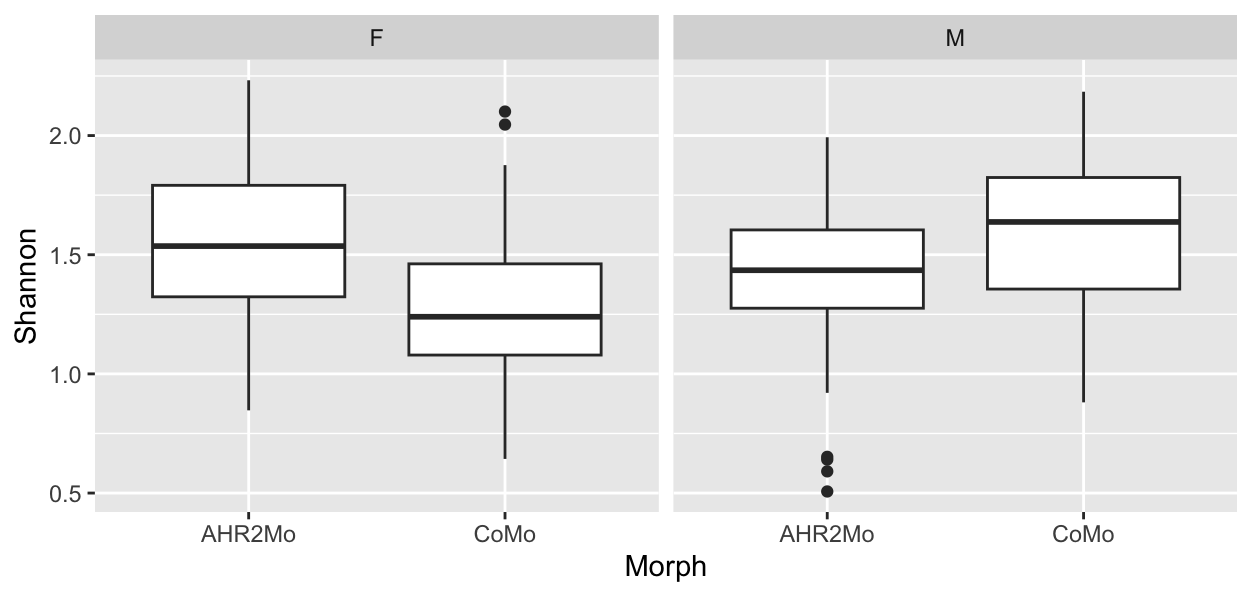

Supplement: Supplementary file 1 [file toxics-13-00010-s001.zip › FigS2.tiff]

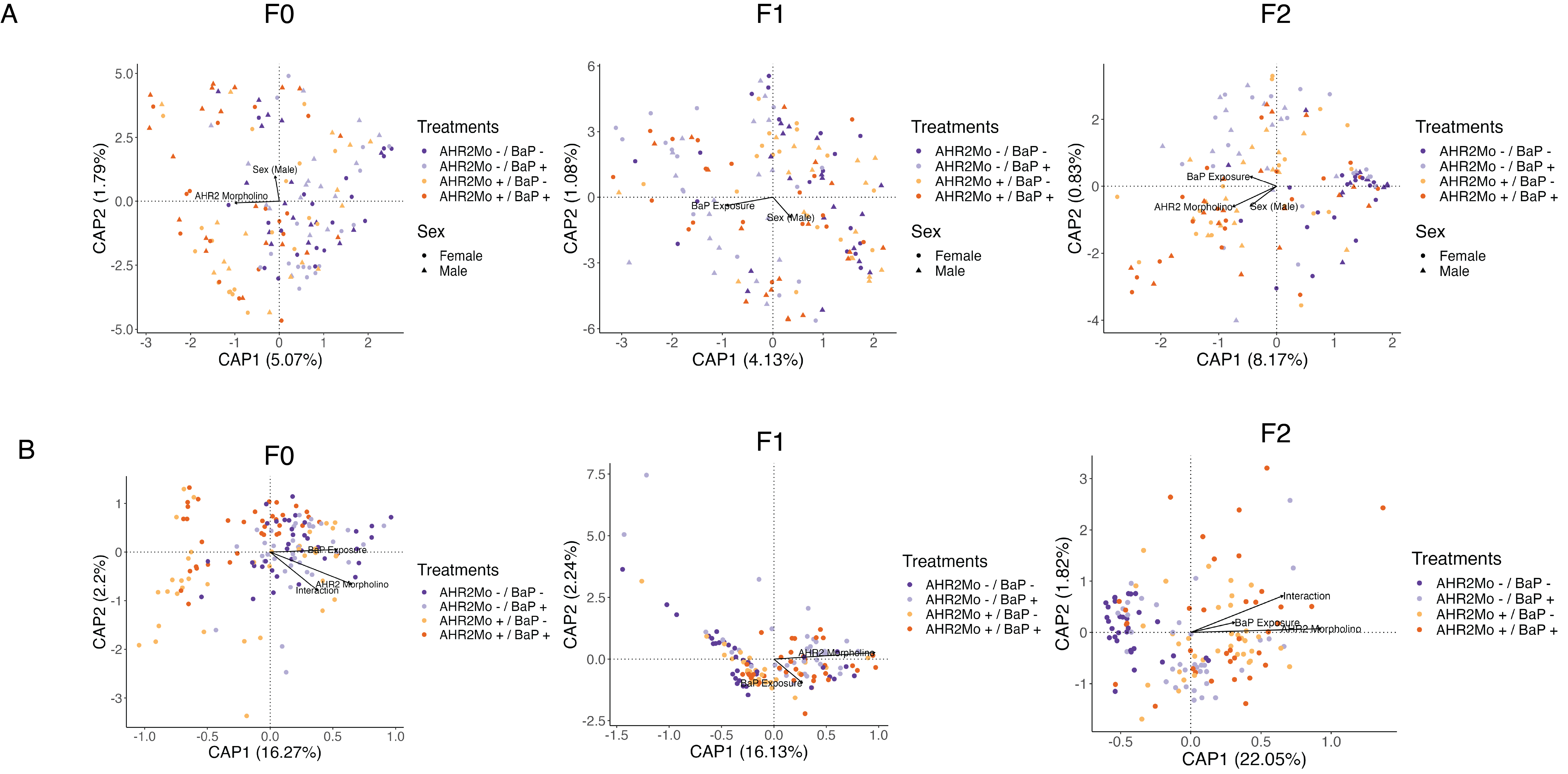

Supplement: Supplementary file 1 [file toxics-13-00010-s001.zip › FigS3.tiff]
